# Testing differential use of payoff-biased social learning strategies in children and chimpanzees

**DOI:** 10.1098/rspb.2017.1751

**Published:** 2017-11-29

**Authors:** Gillian L. Vale, Emma G. Flynn, Jeremy Kendal, Bruce Rawlings, Lydia M. Hopper, Steven J. Schapiro, Susan P. Lambeth, Rachel L. Kendal

**Affiliations:** 1 National Center for Chimpanzee Care, Michale E. Keeling Center for Comparative Medicine and Research, The University of Texas MD Anderson Cancer Center, Bastrop, TX, USA; 2 Department of Psychology and Language Research Center, Georgia State University, Atlanta, GA, USA; 3 Centre for the Coevolution of Biology and Culture, Durham University, Durham, UK; 4 Lester E. Fisher Center for the Study and Conservation of Apes, Lincoln Park Zoo, Chicago, IL, USA

**Keywords:** culture, cultural transmission bias, payoff bias, social learning, social learning strategies

## Abstract

Various non-human animal species have been shown to exhibit behavioural traditions. Importantly, this research has been guided by what we know of human culture, and the question of whether animal cultures may be homologous or analogous to our own culture. In this paper, we assess whether models of human cultural transmission are relevant to understanding biological fundamentals by investigating whether accounts of human payoff-biased social learning are relevant to chimpanzees (*Pan troglodytes*). We submitted 4- and 5-year-old children (*N* = 90) and captive chimpanzees (*N* = 69) to a token–reward exchange task. The results revealed different forms of payoff-biased learning across species and contexts. Specifically, following personal and social exposure to different tokens, children's exchange behaviour was consistent with proportional imitation, where choice is affected by both prior personally acquired and socially demonstrated token–reward information. However, when the socially derived information regarding token value was novel, children's behaviour was consistent with proportional observation; paying attention to socially derived information and ignoring their prior personal experience. By contrast, chimpanzees' token choice was governed by their own prior experience only, with no effect of social demonstration on token choice, conforming to proportional reservation. We also find evidence for individual- and group-level differences in behaviour in both species. Despite the difference in payoff strategies used, both chimpanzees and children adopted beneficial traits when available. However, the strategies of the children are expected to be the most beneficial in promoting flexible behaviour by enabling existing behaviours to be updated or replaced with new and often superior ones.

## 1. Background

Animal culture, defined as behaviour that is socially transmitted, has become the focus of a considerable number of empirical and theoretical studies [1]. Various animals, including cetaceans, primates, fish and birds, exhibit cultures, many of which result in inter-population variation in behavioural repertoires [2]. When researchers began to consider the possibility of culture in non-human animals, the principles of human culture were used as a benchmark. This extension of human cultural attributes to the study of other species has proved fruitful in understanding how organisms negotiate their physical world, revealing important differences in how humans and animals tend to learn from one another, but also some similarities. The broad range of species that acquire information or skills by copying others, or learning from the by-products of others' behaviour, suggests that social learning is a biological fundamental. However, species differ in their propensity to use social information and in the social learning processes they employ to acquire information from others [3–6]. Humans, in particular, show a strong reliance on learning from others [6], whereas many animals use social information solely in situations when collecting personal information is especially costly, obsolete or unreliable [5].

Considerable research effort has been devoted to identifying animal cultures, and to investigating whether homologous mechanisms (e.g. imitation by copying actions) underpin human and other animal cultures. Less well understood are the strategies animals adopt when they use social information [7], despite their role in influencing when and why traits propagate in populations. Various strategies, termed ‘social learning strategies' or ‘cultural transmission biases' [7,8], have been proposed that can determine from whom individuals learn, and when and what to copy. For example, individuals may ‘copy when personal information is outdated', ‘copy when uncertain' or ‘copy knowledgeable individuals' [7,9,10]. The importance of such cultural transmission biases lies in the finding that indiscriminate copying is not always adaptive as it can promote the uptake of maladaptive, unreliable or outdated information [7]. Thus, cultural transmission biases improve fitness through introducing selectivity in when and who to copy, and when to stick to personal information.

Selective use of social learning occurs in various animals, although much work is limited to the investigation of model-based biases, particularly in primate species. Model-based biases are a form of *indirect* bias [8], in that individuals' decisions to copy are influenced by the characteristics of others, rather than the to-be-copied trait itself. Children, for example, preferentially attend to prestigious individuals [11], and copy adults [12], competent models [13] (but see [14]) and accurate models [15] over peers, incompetent models and inaccurate models, respectively. Our evolutionary relatives, chimpanzees, have been shown to preferentially attend to older individuals [16], and copy individuals who are dominant, successful, older and knowledgeable over less dominant, less successful and younger group members [17,18] (although see [19]). Chimpanzees also have attendance biases indicative of ‘copy when uncertain’ and ‘copy when of low rank' strategies [18].

In this paper, we turn our focus to *direct* biases, examining whether the likelihood of copying is affected by trait payoffs. Payoff-biased strategies may be particularly beneficial because the likelihood of copying is related to a direct assessment of the benefit of the observed trait or behaviour (trait payoff), rather than an indirect or model-based bias that can promote maladaptive trait hitchhiking [8,20]. The economist Karl Schlag defined three payoff-biased copying rules that can enable users to adopt fitness maximizing behaviours over repeated learning events (table 1), namely: (i) proportional imitation (PI), where individuals copy the behaviour of another in proportion to how much better the demonstrator's payoff is than the payoff for his/her own behaviour; (ii) proportional observation (PO), where individuals copy in proportion to the value of the demonstrator's payoff using socially acquired information only (here the relative values to self and other are ignored, and copying is determined only according to the value a demonstrator gains for his/her behaviour); and (iii) proportional reservation (PR), also termed ‘copy if dissatisfied', where individuals copy according to the value of their own behavioural payoff only [21,22]. Note that despite its name, proportional *imitation* as defined by Schlag can be underpinned by *any* social learning process and is not restricted to copying of motor patterns.

**Table 1.** Predicted likelihood of use of the socially demonstrated token (*T*_Social_) were individuals to behave according to each of Schlag's three payoff-biased rules (√, likely; X, unlikely; ∼, random). The grey shaded box indicates an omitted condition to minimize required participants. Expressed on the far right are predictions according to the proportion of exchanges of *T*_social_ expected in each of the three reward conditions. (Online version in colour.)

strategy	copy in proportion to	likelihood of using *T*_social_	proportion of *T*_social_ exchanges
proportional imitation (PI)	superiority of model's payoff versus own	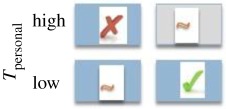	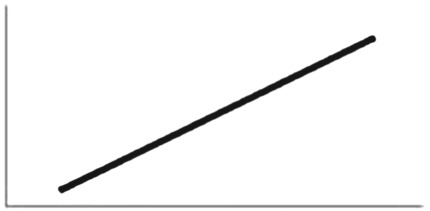
proportional observation (PO)	model's payoff	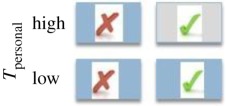	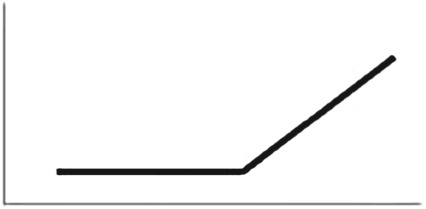
proportional reservation (PR)	satisfaction with own payoff	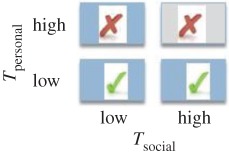	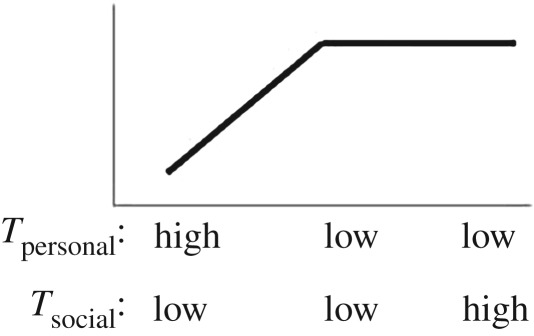

There is indication that some species copy according to one of these payoff contingencies (although see [23]); nine-spined sticklebacks, capuchins and humans alike have been shown to use a PO strategy, with copying dependent upon a demonstrator's payoffs [24–26]. Data for chimpanzees are mixed, however. Some studies suggest chimpanzees rely more heavily on social information when coupled with higher paying rewards, compared with when payoffs to self and other yielded equal rewards [27] (see also [28]). However, chimpanzees also show an overarching tendency to persevere with known behaviour [27] and rely heavily on social information, even when sub-optimal [29]. More recently, chimpanzees have been found to copy efficient task solutions when prior inefficient solutions became difficult to perform, suggesting a form of copying when dissatisfied as payoffs become less frequent [30].

Given that an extension of human models of cultural transmission has been successful in revealing important insights into the social lives of other species (e.g. identifying animal culture), we tested whether Schlag's model of human cultural transmission may be relevant to describing the behaviour of chimpanzees. We compared their behaviour to 4- and 5-year-old children, who are adept social learners and copy selectively (using various model biases [6,11–15]), yet are untested regarding payoff-biased copying. Both species were tested in either a familiar group setting or individually, employing a variant of the token exchange paradigm (see [31]), in which two token types are presented that can be exchanged for rewards. Tokens differed in their outward appearance (contrasting shape and colour) and reward value (high or low value, depending on condition). First we ran a priming experiment to establish whether chimpanzees and children can use payoff-biased strategies following exposure to personal and social token–reward information. Groups of chimpanzees and children first had an opportunity to learn for themselves an association between a token, *T*_personal_, and its reward value (high or low). This was followed by a social prime, exposing them to a trained conspecific demonstrating the exchange of a different token, *T*_social_, and its reward value (high or low). The effect of these primes was measured during the test (open-diffusion) phase, where both types of token were available for exchange. In a second experiment, we investigated payoff-based copying when use of a novel token-type spreads through a population spontaneously, with no demonstrator observation phase. We also ran asocial control conditions, where isolated individuals were not exposed to a demonstrator.

We varied *T*_social_ and *T*_personal_ rewards (high or low) across conditions to discriminate between behaviour consistent with each of Schlag's rules (summarized in table 1). As human adults have been shown to use a PO strategy, we predicted that this strategy may also be evident in early childhood. As chimpanzees display conservative tendencies toward known behaviours [6,27], we predicted they would copy others only when dissatisfied with the payoff to self (PR).

## 2. Material and method

### (a) Participants

Sixty-nine group-living chimpanzees at the NCCC in Texas (USA) participated (*M*_age_ = 29.96 years; 40 females; group sizes range from 5 to 11): 45 in experiment 1 (*N* = 6 groups; seeded with medium-high ranking trained models [29]); 19 in experiment 2 (*N* = 3 groups); and 5 asocial controls (table 2). Ninety children (aged 4- and 5-years; 54 females) participated and were tested in their primary schools (five UK schools) in mixed-sex groups (*N* = 7–10): 51 in experiment 1 (*N* = 6 groups; seeded with female trained models); 30 in experiment 2 (*N* = 3 groups); and 9 asocial controls.

**Table 2.** The number of individuals that participated in each reward condition according to the value (high/low) of the *T*_personal_ and *T*_social_ token, in experiment 1 (2 groups) and experiment 2 (1 group), for (*a*) chimpanzees and (*b*) children. (Online version in colour.)

	
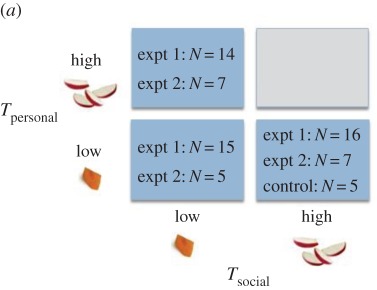	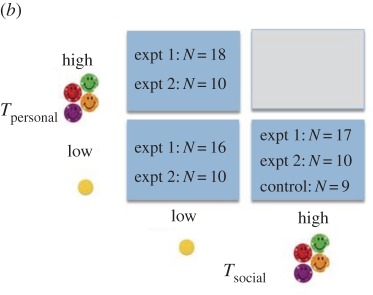

### (b) Materials and procedure

For chimpanzees, we used two types of polyvinyl chloride pipes as non-edible tokens for exchange: black elbow pipes (1.9 cm diameter, height 7.5 cm) and yellow straight pipes (1.9 cm diameter, length 20 cm). For children, we used pipe cleaners as tokens: black (full length, 28 cm) and white (folded in half, 14 cm). Tokens were placed in two correspondingly coloured and spatially segregated opaque token receptacles attached to the mesh of the enclosure (chimpanzees) or placed on the floor (children). Which token colour represented the initially learned token–reward and the side (left/right) on which they were presented was counterbalanced across groups.

For any token exchanged, the experimenter delivered to the participant the corresponding reward (table 2). Rewards were contained in two opaque containers and for chimpanzees consisted of one carrot piece (‘low' value) or four apple pieces (‘high' value) (rewards approximately 2.5 × 2.5 × 0.5 cm). Rewards for children were a single, small, coloured, circular sticker (low) or four larger, circular, sparkly, smily face stickers (high). Three reward conditions, dictating the values of *T*_personal_ and *T*_social_, were presented (table 2): *T*_personal-high_ followed by *T*_social-low,_ *T*_personal-low_ followed by *T*_social-low,_ and *T*_personal-low_ followed by *T*_social-high_.

In both experiment 1 and 2, participants were either tested in groups (social treatment) or individually (asocial control). Chimpanzees were tested in their large outdoor enclosures for the group testing, while asocial controls were tested indoors. For children, the group testing was in a classroom with a teacher present and asocial controls were tested in a separate room or in the school corridor in view of a teacher.

For experiment 1, testing occurred in three stages: (i) personal experience phase, wherein participants gained personal experience exchanging one token type (*T*_personal_ available only) with the experimenter for reward; (ii) model observation phase, wherein groups observed a familiar female (who participated in the personal-experience stage) trained to exchange a novel token type (*T*_social_) (see also electronic supplementary material; note that the experimenter only exchanged tokens with the model during this phase); and (iii) open diffusion test phase, wherein both token types were available to all (30 of each type replenished before depletion). Experiment 2 followed the same procedure omitting the model observation phase. Asocial controls allowed assessment of whether social information influenced token selection, and were tested away from their group, in the key reward condition of *T*_personal-low_ followed by *T*_social-high_.

Chimpanzees were exposed to 3–5 personal-experience sessions (lasting 1 h, experiments 1 and 2) and model observation (lasting 30 min, experiment 1 only) sessions, until 60% of individuals exchanged 20 tokens or observed at least 10 model exchanges (or five sessions had occurred). Cutoff points avoided some participants obtaining extensive personal or social information, while others did not. Model observation sessions were shorter than the personal experience phase to (i) minimize the potential for participants employing a ‘copy when personal information is outdated' strategy [32] and (ii) lessen the likelihood that individuals would copy the model's token preferences irrespective of token payoffs [29] (additional model exposure may strengthen biases towards copying dominant individuals). For the open diffusion phase six 1 h sessions occurred (1 per day). Asocial controls received three personal experience sessions of 15 min and two 20 min test sessions with both tokens available.

Pilot tests with a group of nine children indicated the need to reduce test times to maintain motivation levels. The personal experience phase was 20 min long, followed by 10 min of model observation (experiment 1 only), followed by 30 min of open diffusion with both tokens accessible (minimum two-h between phases). Asocial controls sessions were 10 min long (5 min personal experience and 5 min test).

Groups were randomly assigned to experimental and reward conditions. Asocial controls were individuals that a teacher or care staff member indicated would work individually.

### (c) Data scoring and reliability

Exchanges, token type, exchanger identity, time of exchange and conspecifics attending to it (within 3 m proximity and head orientated towards exchanger/experimenter) were recorded from video. An independent coder assessed a subset of the videos (20 min per reward condition), recording token type exchanged per individual, with high agreement (*κ* coefficient: 0.84, *p* < 0.001).

### (d) Statistical analysis

Models were run using McElreath's Bayesian *rethinking* R package [33]. We constructed multi-level models and generated posterior estimates using *rstan* package's Hamiltonian Monte Carlo. The response variable during the test (open diffusion) phase was either the binomially distributed frequency of each token type exchanged (*T*_personal_ or *T*_social_) or the Poisson distributed number of observations of *T*_social_ exchanges prior to each individual's first *T*_social_ exchange. We constructed a ‘Schlag rules' model which included the following predictor variables, each with an associated coefficient (slope), *β*: sex (male coded 1/female coded 0); the *T*_personal_ reward state (high coded 1/low coded 0); and the *T*_social_ reward state (high coded 1/low coded 0). The Schlag rules model also included separate intercepts (with normally distributed hyperparameters) for individuals (experiments 1 and 2) and social groups (experiment 1). Using the Watanabe–Akaike information criterion (WAIC) as a measure of out of sample deviance, we compared the Schlag-rules model against a null model, which only included the intercepts representing the multi-level structure (i.e. effectively the low personal and social reward state). While it is possible to include species as a predictor variable, we considered each species separately to keep the models simple; consequently our comparison of species is based on interpretation of within-species results rather than a direct statistical evaluation of species difference. We quote the posterior mean, standard deviation and the highest posterior density interval (89% HPDI) for relevant predictor variable coefficients, *β*, in units of log-odds (negative and positive effects of the predictor variable in relation to the response variable lie either side of zero).

## 3. Results

### (a) Experiment 1

#### (i) Children

**Figure 1.**
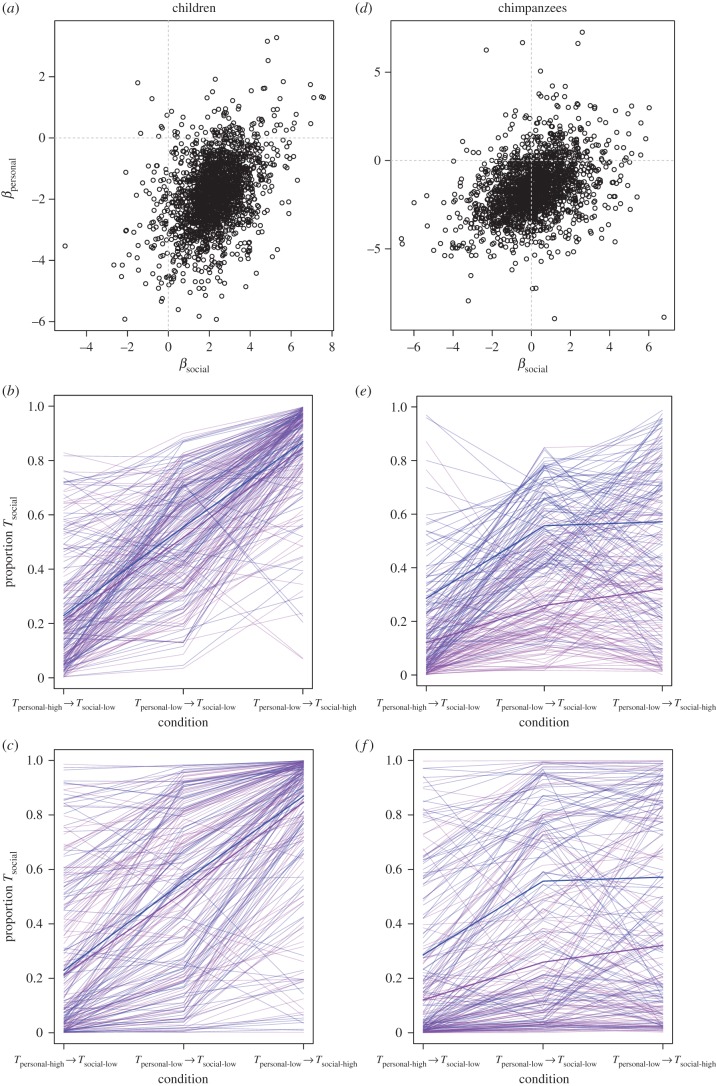
Parts (*a*–*c*) relate to children, while parts (*d*–*f*) relate to chimpanzees in experiment 1. Parts (*a*) and (*d*) show samples from the posterior distribution of coefficient values associated with the personal and social predictor variables. The cloud of points is consistent with the *β* mean, s.d. and HDPI values reported in the text. The cross-hairs distinguish positive and negative values. Parts (*b,c*) and (*c,d*) show the predicted proportion of *T*_social_ exchanges during the test phase by simulated individuals whose behaviour is generated by sampling from the posterior distribution of the Schlag rules model. In parts (*b*) and (*e*) each individual simulation samples from the posterior distribution for an average intercept. By contrast, in parts (*c*) and (*f*), each individual simulation samples from the posterior distribution for an average intercept and in addition, from the posterior variation in individual- and group-level intercepts. Blue lines represent females and purple lines males. The thick lines show the behaviour of an average simulated individual. For each simulated individual, sampled parameter values are held constant across the three conditions indicated on the horizontal axis. (Online version in colour.)

First we considered the frequency of each token type exchanged (*T*_personal_ or *T*_social_) in the test phase. The null model and the Schlag rules model returned similar out-of-sample prediction scores (Schlag rules model WAIC weighting: 51%). However, the standard error for the difference between the two WAIC scores was greater than their difference (dWAIC = 0.1; dSE = 1.01), and given this uncertainty regarding which model was best, the Schlag rules model warranted further investigation. There was no clear effect of sex (*β*_sex_ mean: −0.24; s.d.: 0.44; HDPI: −0.98 to 0.43), a negative effect of the high over low *T*_personal_ condition (*β*_personal_ mean: −1.71; s.d. = 1.20; HDPI: −3.48 to 0.17), and a positive effect of the high over low *T*_social_ condition (*β*_social_ mean: 2.36; s.d. = 1.29; HDPI: 0.36 to 4.34). This suggests children's use of the PI strategy (but see below) as individuals were most likely to use the demonstrated *T*_social_ token if their *T*_personal_ reward was low and the demonstrated reward (*T*_social_) high (figure 1*a*). In addition to the positive and negative clustering of sampled *β*_social_ and *β*_personal_ values, respectively, as illustrated in figure 1*a* there was a positive relationship between *β*_personal_ and *β*_social_ (correlation coefficient, *r* = 0.46). This indicates that although ‘on average' there was evidence consistent with PI, prior exposure to either high reward token (*T*_personal_ or *T*_social_) encouraged future use of the novel *T*_social_ token during test.

Next, we simulated out-of-sample individual behaviour by sampling from the posterior Schlag rules model to illustrate the predicted effect of the posterior distributions in the model on behaviour. As illustrated in figure 1*b*, there was a trend consistent with PI (also table 1) and, as expected, there was no clear sex difference. The larger variance among (simulated) individuals in figure 1*c* versus 1*b* illustrates that there was a substantial effect of individual- and group-level differences (independent intercepts) on variation in predicted behaviour. We note that these two levels had a similar magnitude of effect on the simulated variation (e.g. the standard deviations of their normal distributions were similar), suggesting a considerable proportion of variation in token choice behaviour was attributable to individual and group differences rather than the token–reward condition.

As the *T*_personal_ and *T*_social_ rewards during priming were retained during the test phase, we cannot rule out the possibility that our results were caused by asocial token–reward reinforcement during the test. For example, an individual may simply have tried out both tokens and, as a result of (asocial) reinforcement, shown a general preference for the highly rewarded *T*_social_ token. To test for an effect of social learning as opposed to asocial reinforcement, we compared asocial controls, who received a *T*_personal-low_ prime but no *T*_social-high_ prime in isolation, against individuals from the corresponding group condition who received a *T*_social-high_ prime (controlling for test time: social groups were tested for longer periods, thus it was necessary to cap their exchange time so they were equivalent to asocial controls test durations). An effect of the social priming over and above asocial reinforcement during the test phase would be evident if individuals in the social condition exchanged more of the *T*_social_ tokens than asocial control individuals. We found that the null model, with only variation in intercepts among individuals, and the full model, which also included the two predictor variables, sex and asocial/social condition, performed equally well (full model WAIC weighting: 51%), but note the high uncertainty (dWAIC = 0.1, dSE = 0.39). For the full model, there was no clear effect of sex (*β*_sex_ mean: −1.35; s.d.: 2.74; HDPI: −5.46 to 2.83), and weak evidence that individuals in the social condition were more likely to exchange *T*_social_ during the test phase than those in the asocial condition (*β*_asocial/social_ mean: 1.59; s.d.: 2.25; HDPI: −1.80 to 5.29). We interpreted this as weak evidence of a social influence over and above a possible effect of asocial reinforcement, providing limited support for the original PI result established above.

#### (ii) Chimpanzees

Considering the frequency of each token type exchanged (*T*_personal_ or *T*_social_) as the response variable, the null model and Schlag rules model returned similar out-of-sample prediction scores (Schlag rules WAIC weighting: 40%) and the standard error for the difference between the two WAIC scores was greater than their difference (dWAIC = 0.8; dSE = 1.89), indicating it would be premature to dismiss the Schlag rules model, which revealed an effect of sex (*β*_sex_ ‘mean: −1.48; s.d.: 0.66; HDPI: −2.51 to −0.48), such that females (coded zero) were more likely to exchange *T*_social_ than males (coded one). There was some evidence for a negative effect of the high over low *T*_personal_ priming condition (*β*_personal_ mean: −1.65; s.d. = 1.50; HDPI: −3.96 to 0.84), but no evidence for an effect of the high over low *T*_social_ priming condition (*β*_social_ mean: 0.13; SD = 1.51; HDPI: −2.24 to 2.49; see figure 1*d*), consistent with chimpanzees using PR. Next, we sampled from the posterior Schlag rules model to simulate out-of-sample behaviour. As illustrated in figure 1*e* there was a trend consistent with PR and, on average, females, were more likely to exchange *T*_social_ than males. A comparison of figure 1*e* and 1*f* illustrates that separate intercepts at the individual and group levels had a considerable impact, of similar magnitude, on the variation in the pattern.

As for the children, we compared the social and asocial control conditions on the proportion of each token type exchanged during the test phase. The null model (individual intercepts only) and the full model (individual intercepts and slopes for sex and social/asocial condition) performed equally well (full model WAIC weighting: 53%), but with high uncertainty (dWAIC = 0.2, dSE = 0.65). For the full model, there was no clear effect of sex (*β*_sex_ mean: −1.18; s.d.: 2.66; HDPI: −4.85 to 3.04), and no clear evidence that those in the social learning condition were more likely to exchange the *T*_social_ token than asocial controls, as the standard deviation was high (*β*_asocial/social_ mean: 1.73; s.d.: 2.08; HDPI: −1.52 to 4.83). This lack of response to the social information is consistent with PR in which individuals were most likely to use the demonstrated *T*_social_ token if their *T*_personal_ reward was low in value.

### (b) Experiment 2

By removing the social demonstration phase, this second experiment responded to the concern that in natural diffusions, cues may not be as salient as in experiment 1. Here, individuals only had the opportunity to learn the *T*_social_ reward association once they, or conspecifics, started using it. Thus, if individuals learned a preference for *T*_social_ late in the diffusion process, they would have had less opportunity to preferentially exchange that token compared with those that adopted that preference earlier on. Accordingly, we examined the number of *T*_social_ reward exchanges observed by an individual prior to their first *T*_social_ exchange, which presumably is inversely correlated with the probability of exchanging the *T*_social_ token. Importantly, this variable was a proxy for social influence that cannot be explained by asocial reinforcement learning during test (as it is occurred prior to token exchange).

#### (i) Children

**Figure 2.**
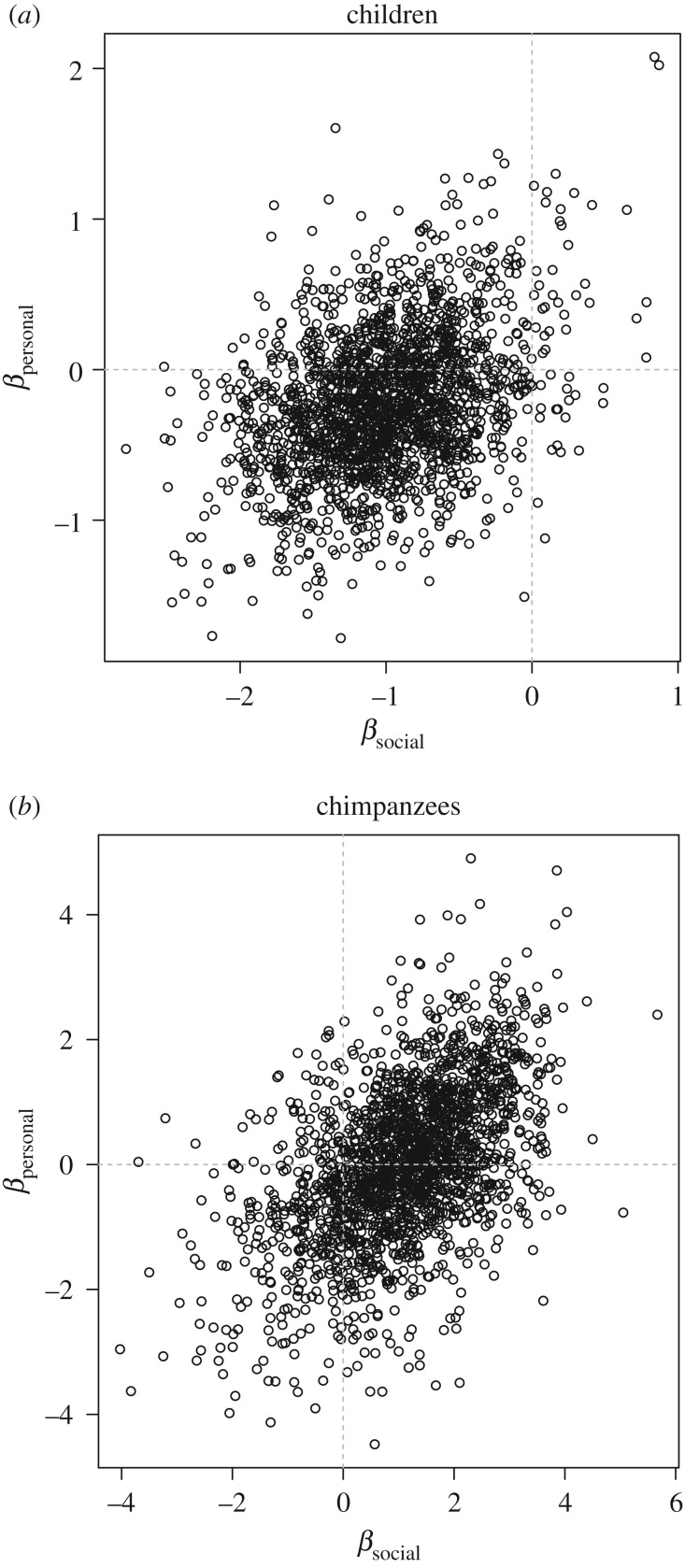
Samples from the posterior distribution of coefficient values associated with the personal and social predictor variables in (*a*) children and (*b*) chimpanzees in experiment 2. The cross-hairs distinguish positive and negative values.

The out-of-sample predictive value of the Schlag rules model (WAIC weighting: 57%) did equally well as the null model, but with considerable uncertainty (dWAIC = 0.6, dSE = 3.3). When interpreting the Schlag rules model, we found no clear effect of sex (*β*_sex_ mean: −0.36; s.d.: 0.46; HDPI: −1.10 to 0.34) or of the *T*_personal_ prime (*β*_personal_ mean: −0.15; s.d. = 0.49; HDPI: −0.98 to 0.56), and a negative effect of the high over low *T*_social_ reward (*β*_social_ mean: −0.99; s.d. = 0.52; HDPI: −1.80 to −0.13). This result indicated that individuals took fewer observations of *T*_social_ before exchanging *T*_social_ tokens for themselves when *T*_social_ returned a high reward compared to low reward, consistent with PO. As in experiment 1, we also observed a weak positive correlation (*r* = 0.36) between *β*_personal_ and *β*_social_ (see figure 2*a*); either personal or social exposure to a high reward encouraged observation of the novel stimulus during the test phase.

#### (ii) Chimpanzees

The out-of-sample predictive value of the Schlag rules model (WAIC weighting: 28%) was less than the null model but with considerable uncertainty (dWAIC = 1.8; dSE = 1.43). Examining the Schlag rules model coefficients, we found no clear effect of sex (*β*_sex_ mean: −0.34; s.d.: 1.14; HDPI: −2.02 to 1.48) or *T*_personal_ priming (*β*_personal_ mean: −0.12; s.d. = 1.17; HDPI: −1.56 to 2.08), and slight evidence for a positive effect of the high over low *T*_social_ value (*β*_social_ mean: 1.20; s.d. = 1.28; HDPI: −0.80 to 3.21). Keeping in mind the considerable uncertainty concerning the latter result, it is possible that chimpanzees observed more *T*_social_ exchanges before exchanging their first *T*_social_ token when *T*_social_ returned a high over low reward. This may represent an attentional bias towards high value food items. We also noted a positive correlation (*r* = 0.52) between *β*_personal_ and *β*_social_ (see figure 2*b*.

## 4. Discussion

We examined whether chimpanzees and 4- and 5-year-old children strategically copied a novel behaviour (token choice) depending on the difference in payoff between the individual's current and new behaviour. The results provide some evidence that children are capable of PI when first exposed to personal followed by social information, prior to test (experiment 1). But if the socially derived information was novel at the start of the test phase (experiment 2), children appeared to respond only to the reward value of that novel token, and were unaffected by prior personal information. This suggests children's use of a PO strategy, in which the probability of copying a novel behaviour depends upon the socially demonstrated reward value only. By contrast, the chimpanzees showed no clear evidence of social learning and behaved according to PR, relying on their prior information to guide token choice during test, although with some evidence of attentional bias towards high-rewarding conspecific exchanges (experiment 2). As there is clear evidence that chimpanzees can learn socially [4], our results emphasize that the degree to which they are actually affected by social stimuli appears to be context dependent.

In experiment 1, we also find a sex difference in chimpanzees for the probability of switching to a new behaviour. Specifically, females, overall, exchanged more of the socially demonstrated token than did male chimpanzees. This may suggest females are less neophobic (or more exploratory) than males, and males may be more conservative than females, in persevering with a familiar learned behaviour.

Chimpanzees have recently been found to persevere with costly and inefficient task solutions despite conspecific demonstrations of quick and easy alternatives [27], and only when inefficient solutions become difficult to perform do chimpanzees generally adopt the socially demonstrated efficient behaviour [30]. One interpretation of these findings is that chimpanzees are inclined to copy others when dissatisfied with the payoffs associated with the known behaviour, as they either become less frequent [30] or are of low value (current study). This use of PR indicates that conservative tendencies in chimpanzees [6,27] may not always reflect difficulty in forgoing a known solution per se, but rather, may reflect a lack of motivation to adopt new behaviours if a known behaviour is sufficiently rewarding (see [28]).

Where payoffs to behaviours differ in magnitude or quality, individuals may be more or less prone to explore the behaviours available to them. We saw this in both chimpanzees and children, who showed some inclination to exchange the novel token when either their personal or social token yielded a high reward. This may suggest that the mere presence of high rewards affects behaviour, leading to an exploration of the task parameter space (i.e. individuals explore the alternative options available to them). Social facilitation, in which the presence of other individuals increases individual activity, is well documented in animal species [34] (reviewed in [35]) and has been proposed to lead indirectly to social learning as audience effects increase the likelihood that individuals adopt exploratory behaviour due to reduced neophobia [35]. Capuchin monkeys, for example, have been found to sample more of a novel food when in the presence of other individuals, relative to solo control conditions [36]. Our results add to this by indicating that social facilitation effects may also relate to the reward values involved. In particular, the presence of preferred rewards may increase individuals' exploratory behaviour, perhaps through the effect they have on arousal or motivation levels.

Our analyses reveal large individual- and group-level variation in both species, as evident from the effect of their intercepts in our out-of-sample predictions. Moreover, our results are specific to the developmental and cultural context of our participants. This indicates that further work is needed to identify what is affecting individual and cultural variation [37,38]. We note that while the uncertainty in our results warrants caution, it may be indicative of simultaneous use of multiple strategies (e.g. [18,24]). Indeed, the results of experiments 1 and 2 suggest that children use different payoff strategies according to context. Specifically, in a direct test of the Schlag rules (experiment 1) we found evidence that children used PI. By contrast, when focusing on the amount of social information collected prior to adopting a behaviour (experiment 2), the children's behaviour was consistent with PO. This indicates that multiple strategies can be used, dependent upon the conditions individuals are exposed to.

## 5. Conclusion: are humans a good model for other animals?

The aim of our study, in line with the topic of this special issue, was to compare whether humans and chimpanzees used the same payoff bias. In the context of our experiment, employing very similar tasks across species, we found no indication of similarity in response despite chimpanzees constituting one of our closest living relatives. This may be taken as evidence that human models of cultural transmission have very little use for our understanding of the social lives of other species. However, with the goal of comparative and evolutionary psychology in mind, it is only by comparing humans and other animals that we may glean important insights into both similarities and differences between species. Without such comparisons, deciphering the evolutionary trajectory of psychological attributes is extremely difficult. Thus, the interchange of information from those who work with animals and humans continues to play a vital role in identifying shared traits, as well as specialisms that distinguish species.

## Supplementary Material

Additional Detail of Methods & Raw Data

## Data accessibility

All raw data are available in the electronic supplementary material.
