# Supplementary material for: Testing differential use of payoff-biased social learning strategies in children and chimpanzees
Source: Proc Biol Sci. 2017 Nov 29;284(1868):20171751. doi: 10.1098/rspb.2017.1751 (PMC5740275; doi:10.1098/rspb.2017.1751)
Supplement: Additional Detail of Methods & Raw Data [file rspb20171751supp1.pdf]

Electronic supplementary materials

Testing differential use of payoff-biased social learning strategies in children and chimpanzees

Gillian L. Vale, Emma G. Flynn, Jeremy Kendal, Bruce Rawlings, Lydia M. Hopper, Steven J. Schapiro, Susan P. Lambeth & Rachel L. Kendal

1. **Methods**

(a) Reward Preferences.

Dichotomous food preferences were initially run to identify preferred food quantities. Preference tests involved baiting two feeder tubes with food, differing in quantity, (alternating the side on which the large quantity was placed) and then presenting them simultaneously to chimpanzees, allowing a forced choice. Pilot tests with 1vs4 grapes (*N*=5) and 1vs8 (*N*=6) grapes failed to provide consistent preferences. Dichotomous preference tests with food differing in both quality and quantity (1 carrot piece versus 4 apple pieces) did yield consistent preferences (for apple), defined as selection of one food type on 8 or more of the 10 trials, in all but one participant. As this individual was an asocial control, food preference tests were run with a different food item (1 carrot piece versus 4 green pepper pieces) and yielded a consistent preference (for pepper). This alternative food was used in subsequent tests for this individual. All preference tests were run on different days to the main study.

(b) Exchanging for carrot.

A pilot test was conducted with individuals not participating in the main study (*N*=7) to ensure that chimpanzees were sufficiently motivated to exchange a token for the less preferred reward (carrot). The test consisted of dispensing multiple tokens into chimpanzees' outdoor enclosure. An experimenter stood in a location approximately 3.96 metres away from the area in which the tokens were dispensed, with the right arm extended towards the chimpanzees enclosure (palm-up beg gesture). All chimpanzees exchanged tokens for single carrot pieces (range 4-21, across a 20 minute period). Thus carrot was considered sufficiently motivating for token exchange.

(c) Token-Colour preferences.

Preference tests were also conducted to determine whether individuals displayed a bias towards one of the two token colours. The experimenter held one token in each hand, arms outstretched towards the participant, allowing a forced choice to be made by gesture. A preference was considered present if one token type was selected on 8 (or more) of 10 trials. No participant displayed a token preference. No rewards were provisioned during token preference tests and all token preference tests were conducted on different days to experimental tests, often over multiple days/sessions where participant motivation was low.

(d) Model Training.

For chimpanzees, models were trained in isolation, out of view of their group members. Medium to high dominance bounds were used for model selection as the highest ranking female in each group would not always voluntarily isolate for training (or be motivated to train). Models were trained to exchange one token type by only rewarding exchanges with the desired token. Initially only the desired token was made available forcing the participant to exchange this token only. Multiple tokens of each type were then provisioned. Models were considered trained upon exchanging the desired token, when both were available, 10 consecutive times across two sessions. Each training session lasted between ca. 15-30 minutes.

For children, one female (medium dominance/popularity/age) from each group served as the model. Dominance and popularity was assessed by two members of staff using ratings (who, in each dyad, would win a contest over a toy [dominance] and who had more friends in their class

[popularity]. Model training consisted of simply asking the model, away from the rest of the group, to exchange the pre-specified token. Understanding was confirmed by requesting the model to repeat what they had been asked and by asking ‘what token should you exchange?’

(e) Model observation phase

Sessions would begin when the model approached the exchange area. If necessary, the experimenter would call the model over to this area. The alternative token was handed to the model by the experimenter. To gain a reward, the model had to take the token and return it to the experimenter. All group members were free to observe model exchanges. Token provisioning was employed to control who received the token (i.e. model only), thus ensuring only social information was available. Token receptacles were not baited as it was deemed unlikely that models were of sufficient dominance to gain access to a single token if placed in its receptacle. If conspecifics stole the alternative token from the model, the exchange was rewarded to avoid extinguishing the behaviour.

2. Raw Data

When viewing these data note that for:

‘Reward Conditions’

SL = $T_{personal-}$low followed by $T_{social-}$high

SS= $T_{personal-}$low followed by $T_{social-}$low

LS= $T_{personal-}$high followed by $T_{social-}$low

‘Model Condition’

Asocial = Asocial control individuals

Seeded = Experiment 1

Non-seeded = Experiment 2

‘First Token type exchanged’

Pretrained = $T_{personal}$

Alternative = T_{social}

Reward Condition	Model Condition	Species	Sex	Group	Personal Experience (pre-experienced token exchanges in personal experience phase)	Prop Alt Exchanged Open Diffusion
SL	Asocial	Chimpanzee	F	Asocial	68	0
SL	Asocial	Chimpanzee	M	Asocial	64	0
SL	Asocial	Chimpanzee	M	Asocial	53	0.5
SL	Asocial	Chimpanzee	M	Asocial	53	0
SL	Asocial	Chimpanzee	M	Asocial	77	0.72
SL	Asocial	Child	F	Asocial	21	0
SL	Asocial	Child	F	Asocial	26	0.45
SL	Asocial	Child	F	Asocial	21	0.49
SL	Asocial	Child	M	Asocial	20	0.76
SL	Asocial	Child	M	Asocial	24	0.95
SL	Asocial	Child	F	Asocial	20	0.86
SL	Asocial	Child	F	Asocial	18	0.59
SL	Asocial	Child	F	Asocial	20	0.48
SL	Asocial	Child	M	Asocial	15	0

Reward Condition	Model Condition	Species	Sex	Group	1st Token type exchanged	Personal Experience (pre-experienced token exchanges in personal experience phase)	Alt observations prior to first exchange (non-seeded condition)	Prop Alt exchanged after witnessing a conspecific exchange the Alt	Prop Alt Exchanged Open Diffusion
SS	Non-Seeded	Child	F	G1	alternative	9	0	1	1
SS	Non-Seeded	Child	F	G1	alternative	5	2	0.86	0.86
SS	Non-Seeded	Child	M	G1	alternative	10	2	0.7	0.7
SS	Non-Seeded	Child	F	G1	pretrained	10	2	0.5	0.5
SS	Non-Seeded	Child	M	G1	alternative	14	0	0.3	0.33
SS	Non-Seeded	Child	F	G1	pretrained	6	9	0.88	0.88
SS	Non-Seeded	Child	F	G1	pretrained	11	2	0.82	0.76
SS	Non-Seeded	Child	F	G1	pretrained	8	12	0.13	0.13
SS	Non-Seeded	Child	M	G1	alternative	15	7	1	1
SS	Non-Seeded	Child	F	G1	pretrained	18	6	0.5	0.5
LS	Non-Seeded	Child	F	G2	alternative	17	1	0.59	0.59
LS	Non-Seeded	Child	M	G2	pretrained	7		0	0
LS	Non-Seeded	Child	M	G2	alternative	9	2	0.18	0.15
LS	Non-Seeded	Child	F	G2	alternative	9	0	0	0.04
LS	Non-Seeded	Child	F	G2	alternative	1	3	1	1
LS	Non-Seeded	Child	F	G2	alternative	13	3	0.59	0.59
LS	Non-Seeded	Child	M	G2	alternative	4	4	0.59	0.59
LS	Non-Seeded	Child	F	G2	pretrained	10	8	0.41	0.41
LS	Non-Seeded	Child	F	G2	alternative	14	3	0.05	0.05
LS	Non-Seeded	Child	F	G2	pretrained	7		0	0
SL	Non-Seeded	Child	M	G3	pretrained	10	2	1	1
SL	Non-Seeded	Child	F	G3	pretrained	10	1	0.94	0.94
SL	Non-Seeded	Child	F	G3	alternative	9	2	0.88	0.88
SL	Non-Seeded	Child	M	G3	pretrained	10	0	1	0.94
SL	Non-Seeded	Child	M	G3	alternative	9	1	0.82	0.82
SL	Non-Seeded	Child	M	G3	pretrained	5	0	0.95	0.92
SL	Non-Seeded	Child	F	G3	alternative	10	2	1	1
SL	Non-Seeded	Child	M	G3	alternative	9	1	1	1
SL	Non-Seeded	Child	F	G3	alternative	9	2	1	0.9
SL	Non-Seeded	Child	M	G3	alternative	8	1	1	1
SS	Seeded	Child	F	G4	alternative	18		0.44	0.44
SS	Seeded	Child	F	G4	pretrained	16		0.81	0.81
SS	Seeded	Child	F	G4	alternative	14		0.53	0.53
SS	Seeded	Child	F	G4	pretrained	15		0.76	0.76
SS	Seeded	Child	M	G4	alternative	16		0.17	0.17
LS	Seeded	Child	F	G5	alternative	12		0.42	0.42
LS	Seeded	Child	F	G5	pretrained	20		0.47	0.47
LS	Seeded	Child	F	G5	alternative	23		0.54	0.54
LS	Seeded	Child	F	G5	alternative	14		0.64	0.64
LS	Seeded	Child	F	G5	alternative	12		0.7	0.7
LS	Seeded	Child	M	G5	alternative	37		0.6	0.6
LS	Seeded	Child	M	G5	alternative	24		0.21	0.21
SL	Seeded	Child	M	G6	pretrained	12		0.92	0.92
SL	Seeded	Child	M	G6	alternative	6		1	1
SL	Seeded	Child	F	G6	alternative	9		0.92	0.92
SL	Seeded	Child	M	G6	alternative	7		1	1
SL	Seeded	Child	M	G6	alternative	4		0.95	0.95
SL	Seeded	Child	M	G6	alternative	13		1	1
SL	Seeded	Child	M	G6	pretrained	14		1	1
SL	Seeded	Child	F	G6	alternative	9		1	1

Reward Condition	Model Condition	Species	Sex	Group	1st Token type exchanged	Personal Experience (pre-experienced token exchanges in personal experience phase)	Alt observations prior to first exchange (non-seeded condition)	Prop Alt exchanged after witnessing a conspecific exchange the Alt	Prop Alt Exchanged Open Diffusion
SS	Seeded	Child	F	G7	alternative	8		0.96	0.96
SS	Seeded	Child	F	G7	alternative	7		1	1
SS	Seeded	Child	M	G7	pretrained	7		0.81	0.81
SS	Seeded	Child	F	G7	alternative	4		1	1
SS	Seeded	Child	M	G7	alternative	7		1	1
SS	Seeded	Child	F	G7	alternative	6		0.11	0.11
SS	Seeded	Child	M	G7	alternative	5		0.59	0.59
SS	Seeded	Child	M	G7	alternative	3		1	1
SS	Seeded	Child	M	G7	pretrained	8		0.32	0.32
SL	Seeded	Child	M	G8	pretrained	8		0.98	0.98
SL	Seeded	Child	F	G8	pretrained	10		0.88	0.88
SL	Seeded	Child	M	G8	alternative	4		0.94	0.94
SL	Seeded	Child	M	G8	alternative	9		0.94	0.94
SL	Seeded	Child	M	G8	alternative	5		0.91	0.91
SL	Seeded	Child	F	G8	alternative	11		0.8	0.8
SL	Seeded	Child	F	G8	alternative	4		0.89	0.89
LS	Seeded	Child	F	G9	alternative	7		0.07	0.07
LS	Seeded	Child	F	G9	alternative	7		0.09	0.09
LS	Seeded	Child	F	G9	alternative	6		0.1	0.1
LS	Seeded	Child	F	G9	alternative	15		0.12	0.12
LS	Seeded	Child	F	G9	alternative	8		0.42	0.42
LS	Seeded	Child	M	G9	alternative	4		0.03	0.03
LS	Seeded	Child	M	G9	alternative	6		0.1	0.1
LS	Seeded	Child	F	G9	alternative	9		0.19	0.19
LS	Seeded	Child	M	G9	alternative	4		0.28	0.28
SL	Non-Seeded	Chimpanzee	F	G10	alternative	97	0	0.62	0.63
SL	Non-Seeded	Chimpanzee	F	G10	alternative	58	3	0.4	0.4
SL	Non-Seeded	Chimpanzee	M	G10	pretrained	2		0	0
SL	Non-Seeded	Chimpanzee	F	G10	pretrained	25	10	0.57	0.57
SL	Non-Seeded	Chimpanzee	M	G10	pretrained	35	0	0.19	0.22
SL	Non-Seeded	Chimpanzee	M	G10	pretrained	36	12	0.37	0.37
SL	Non-Seeded	Chimpanzee	F	G10	pretrained	7		0	0
LS	Seeded	Chimpanzee	M	G11	pretrained	43		0.19	0.19
LS	Seeded	Chimpanzee	M	G11	alternative	74		0.26	0.26
LS	Seeded	Chimpanzee	F	G11	pretrained	22		0.52	0.52
LS	Seeded	Chimpanzee	M	G11	alternative	1		1	1
LS	Seeded	Chimpanzee	M	G11	alternative	20		0.58	0.58
LS	Seeded	Chimpanzee	F	G11	alternative	20		0.58	0.58
SL	Seeded	Chimpanzee	F	G12	alternative	56		0.91	0.91
SL	Seeded	Chimpanzee	F	G12	alternative	5		0.97	0.97
SL	Seeded	Chimpanzee	M	G12	alternative	14		1	1
SL	Seeded	Chimpanzee	M	G12	alternative	24		0.27	0.27
SL	Seeded	Chimpanzee	F	G12	pretrained	31		0.75	0.75
SL	Seeded	Chimpanzee	F	G12	alternative	19		0.87	0.87
SL	Seeded	Chimpanzee	F	G12	pretrained	46		0.32	0.32
SL	Seeded	Chimpanzee	F	G12	pretrained	84		0.7	0.7
SL	Seeded	Chimpanzee	F	G12	alternative	11		0.59	0.59
SL	Seeded	Chimpanzee	F	G12	alternative	7		0.86	0.86

Reward Condition	Model Condition	Species	Sex	Group	1st Token type exchanged	Personal Experience (pre-experienced token exchanges in personal experience phase)	Alt observations prior to first exchange (non-seeded condition)	Prop Alt exchanged after witnessing a conspecific exchange the Alt	Prop Alt Exchanged Open Diffusion
SS	Non-Seeded	Chimpanzee	F	G13	alternative	234	0	0.49	0.5
SS	Non-Seeded	Chimpanzee	F	G13	alternative	212	2	0.28	0.28
SS	Non-Seeded	Chimpanzee	M	G13	alternative	10	1	1	1
SS	Non-Seeded	Chimpanzee	F	G13	alternative	7	0		1
SS	Non-Seeded	Chimpanzee	F	G13	alternative	18	2	1	0.19
LS	Non-Seeded	Chimpanzee	F	G14	pretrained	97	0	0.07	0.07
LS	Non-Seeded	Chimpanzee	F	G14	pretrained	44	1	0.39	0.35
LS	Non-Seeded	Chimpanzee	M	G14	alternative	37	0	0.93	0.93
LS	Non-Seeded	Chimpanzee	M	G14	pretrained	20	4	0.34	0.33
LS	Non-Seeded	Chimpanzee	F	G14	alternative	42	1	0.31	0.31
LS	Non-Seeded	Chimpanzee	M	G14	alternative	1	0		1
LS	Non-Seeded	Chimpanzee	F	G14	pretrained	2	6	0.5	0.5
SS	Seeded	Chimpanzee	M	G15	alternative	217		0.08	0.08
SS	Seeded	Chimpanzee	M	G15	alternative	14		0.6	0.6
SS	Seeded	Chimpanzee	M	G15	alternative	136		0.55	0.55
SS	Seeded	Chimpanzee	M	G15	alternative	64		0.06	0.06
SS	Seeded	Chimpanzee	F	G15	alternative	2		1	1
SS	Seeded	Chimpanzee	F	G15	alternative	2		0.83	0.83
SS	Seeded	Chimpanzee	M	G15	pretrained	2		0	0
SS	Seeded	Chimpanzee	M	G15	pretrained	23		0.08	0.08
LS	Seeded	Chimpanzee	F	G16	alternative	202		0.1	0.1
LS	Seeded	Chimpanzee	M	G16	pretrained	31		0	0
LS	Seeded	Chimpanzee	F	G16	alternative	35		0.12	0.12
LS	Seeded	Chimpanzee	F	G16	pretrained	15		0.14	0.14
LS	Seeded	Chimpanzee	F	G16	pretrained	11		0	0
LS	Seeded	Chimpanzee	M	G16	pretrained	68		0.03	0.03
SL	Seeded	Chimpanzee	F	G17	pretrained	56		0.41	0.41
SL	Seeded	Chimpanzee	M	G17	pretrained	25		0	0
SL	Seeded	Chimpanzee	F	G17	pretrained	37		0.9	0.9
SL	Seeded	Chimpanzee	F	G17	pretrained	33		0.35	0.35
SS	Seeded	Chimpanzee	F	G18	alternative	20		0.79	0.79
SS	Seeded	Chimpanzee	M	G18	alternative			0.54	0.54
SS	Seeded	Chimpanzee	M	G18	pretrained	26		0.1	0.1
SS	Seeded	Chimpanzee	F	G18	alternative	44		0.96	0.96
SS	Seeded	Chimpanzee	M	G18	pretrained	7		0.94	0.94
